# The prognostic value of MicroRNAs associated with fatty acid metabolism in head and neck squamous cell carcinoma

**DOI:** 10.3389/fgene.2022.983672

**Published:** 2022-08-30

**Authors:** Xiaojing Wang, Yue Zhao, Dorothee Franziska Strohmer, Wenjin Yang, Zhijia Xia, Cong Yu

**Affiliations:** ^1^ Department of Neurosurgery, Shanghai Pudong New Area People’s Hospital, Shanghai, China; ^2^ Department of Neurology, The First Affiliated Hospital of Anhui Medical University, Hefei, China; ^3^ Department of Breast Surgery, The Cancer Hospital of the University of Chinese Academy of Sciences (Zhejiang Cancer Hospital), Hangzhou, China; ^4^ Department of General, Visceral, and Transplant Surgery, Ludwig-Maximilians-University Munich, Munich, Germany

**Keywords:** fatty acid metabolism, MicroRNAs, head and neck squamous cell carcinoma, prognostic, risk score

## Abstract

Head and neck squamous cell carcinoma (HNSCC) is the sixth most frequent cancer in humans globally. In addition to smoking and drinking, genetic and epigenetic changes also play a big role in how HNSCC starts and grows. MicroRNAs are short, non-coding RNAs that control cell differentiation and apoptosis by interfering with gene expression. In addition, microRNAs in HNSCC have been shown to affect the clinical behaviors of HNSCC in amazing ways. Moreover, metabolic reprogramming is a key part of cancer and is needed for cancer to turn into a tumor and grow. But it is still not clear what effect microRNAs related to fatty acid metabolism have on the prognosis of HNSCC patients. We downloaded the data of HNSCC patients from the TCGA database and obtained the genes associated with fatty acid metabolism according to the GSEA database. Then, the microRNAs associated with fatty acid metabolism genes were matched. Finally, fatty acid metabolism gene-associated microRNAs for calculating risk scores and then building multifactorial Cox regression models in patients with HNSCC. Heatmap analysis showed that microRNAs involved in fatty acid metabolism were significantly different in HNSCC patients than in healthy controls. A total of 27 microRNAs associated with fatty acid metabolism were screened by univariate Cox analysis (*p* < 0.05). Using lasso regression, 18 microRNAs substantially linked with the prognosis of HNSCC patients were identified and included in risk scores. The ROC curves demonstrate that risk scores derived from microRNAs involved in fatty acid metabolism can accurately predict the prognosis of HNSCC patients at 1, 3, and 5 years. Moreover, we discovered that 11 microRNAs included in the risk score properly distinguished the prognosis of HNSCC patients. This paper indicated that microRNAs involved with fatty acid metabolism are strongly linked to the prognosis of HNSCC patients. It also indicated that reprogramming of fatty acid metabolism in tumor tissues may play an important role in HNSCC cancer.

## Introduction

Head and neck squamous cell carcinoma (HNSCC), one of the most prevalent malignancies in the world, is a cancer of the squamous epithelium that lines the head and neck organs, such as the oral cavity, larynx, hypopharynx, oropharynx, nasopharynx, paranasal sinuses, nasal cavity, and salivary glands with poor outcomes and survival rates (Shiah et al., 2021). However, palliative care is being considered as a systematic therapy for patients with recurrent or spread HNSCC. This places a huge burden on society and the healthcare system. So, it is important to find useful prognostic and predictive biomarkers linked to the clinical prognosis of HNSCC in order to improve the overall survival rate.

In the last 10 years, it has been shown that microRNAs play an important role in the development of HNSCC cancer and its spread (Qiu et al., 2021; Shiah et al., 2021; Yang et al., 2019; Yu et al., 2019). This makes them good candidates for biomarkers that can predict the biological activity of HNSCC. Reprogramming of metabolic pathways ensures the survival and proliferation of cancer cells in a nutrient-deficient environment (Pavlova and Thompson, 2016; Soltani et al., 2021). Changes in tumor cell metabolism and immunosuppression have big effects on how tumors grow. A lot of research has been done on how they control and take over the metabolic reprogramming of immune cells and the tumor microenvironment to keep growing tumors from being attacked by the immune system (Pacella et al., 2018; Siddiqui and Glauben, 2022; Wu L. et al., 2020). A previous study found the inactivation of receptor-interacting protein kinase 3 (RIPK3) in tumor-associated macrophages (TAMs) reprograms fatty acid metabolism via the ROS-caspase1-PPAR pathway, which accelerated HCC development (Wu L. et al., 2020). TAMs can live in places where there is not enough food or oxygen, change their metabolism to become pro-tumorigenic or suppress the immune system, and help tumors grow by turning on the FAS and PPAR pathways (Gajewski et al., 2013; Wu L. et al., 2020).

Lipid metabolism, particularly fatty acid synthesis, is a critical physiological process that turns nutrients into metabolic intermediates for membrane formation, energy storage, and the creation of signaling molecules. A major metabolic characteristic of cancer cells is altered lipid metabolism (Hao et al., 2019). Furthermore, changing lipid availability influences cancer cell motility, angiogenesis development, metabolic symbiosis, immune surveillance evasion, and cancer medication resistance. The reprogramming of fatty acid metabolism in tumor tissues has attracted a lot of attention as a possible cancer therapeutic target (Pacella et al., 2018; Wu J. Y. et al., 2020; Wu L. et al., 2020). Recently, the relevance of tumor metabolism in HNSCC and its surrounding environment has gained more attention. However, there is no study on the predictive value of microRNAs involved in fatty acid metabolism in HNSCC. So, we did this study to find out how well certain microRNAs involved in the metabolism of fatty acids can predict the outcome of HNSCC.

## Methods and materials

### Database

The RNA-sequencing transcriptome data and clinical data on patients with HNSCC and a matched normal group were extracted from the TCGA database (https://tcga-data.nci.nih.gov/tcga/). Ninety-two fatty acid metabolism-related genes were downloaded from the GeneCards (https://www.genecards.org/), including ABCD1, ABCD2, ACAA2, ACAD9, ACADL, ACADM, ACADS, ACADVL, ACAT1, ACSL1, ACSL3, ACSL4, ACSL5, ACSL6, ACSM1, ACSM2A, ACSM2B, ACSM3, ACSM4, ACSS1, ACSS2, ACSS3, CPT1A, CPT1B, CPT2, CRAT, CROT, ECHS1, ETFA, ETFB, ETFDH, HADH, HADHA, HADHB, HSD17B10, PEX11G, PEX13, PEX14, SLC25A20, ACAT2, ACAA1, EHHADH, ACOX3, ACOX1, ACADSB, GCDH, ACSBG1, ACSBG2, CPT1C, ECI1, ECI2, CYP4A11, CYP4A22, ADH1A, ADH1B, ADH1C, ADH7, ADH4, ADH5, ADH6, ALDH2, ALDH3A2, ALDH1B1, ALDH7A1, ALDH9A1, ACACA, ACACB, ACLY, FASN, MCAT, OLAH, OXSM, MECR, PPT1, PPT2, ELOVL1, ELOVL2, ELOVL3, ELOVL4, ELOVL5, ELOVL6, ELOVL7, HSD17B12, HACD2, HACD1, HACD4, HACD3, TECR, ACOT4, ACOT2, ACOT1, ACOT7 (collect and download from GeneCards). MicroRNAs related to these 92 genes were retrieved for further investigation.

This research didn’t have to acquire ethical approval for our study because we used information that was already in the public database.

### Bioinformatics analysis

The expression of fatty acid metabolism-related microRNAs in tumor tissues and matched normal tissues was compared to detect DEGs using the R package (“pacman”, “limma”, “edgeR”, “pheatmap”) in R (https://www.r-project.org/, version R 4.2.1). The ratios for all fatty acid metabolism-related microRNAs were computed between samples to measure the fold-change (FC) in expression between groups. DEG was determined using the following criteria: A false discovery rate (FDR) < 0.05 and a |log2FC| value greater than 1.0 are necessary. The expression of these fatty acid metabolism-related microRNAs was then visualized using vioplot and heatmap.

The univariate Cox regression analysis was used to screen for fatty acid metabolism-related microRNAs associated with HNSCC patients’ outcome (*p* < 0.05). Using 10-fold cross-validation and a *p*-value of 0.05, Least Absolute Shrinkage and Selection Operator (LASSO) regression was conducted. After identifying possible prognostic genes, the risk score was determined using the risk score formula. By using the median risk score as the dividing line, we classified the patients as either low-risk or high-risk. Using the Kaplan-Meier technique and the log-rank test, the overall survival (OS) between the two groups was determined. Risk scores are represented graphically after separating high and low risk categories. Risk scores are represented graphically. Using the “survival,” “survminer,” and “timeROC” functions of the R package, the 1-, 3-, and 5-year time-dependent receiver operating characteristics (ROC) curves were shown. Using microRNAs associated with fatty acid metabolism that were filtered by lasso regression, multivariate Cox regression models were built, and forest plots were generated. The univariate Cox regression and multivariable Cox regression were used to explore whether the risk score based on fatty acid metabolism-related microRNAs and characteristics of patient and tumor (age, gender, grade of tumor differentiation, and TNM stage) were independent risk factors and to construct prognostic Norman diagrams based on the above characteristics. The log-rank test of the Kaplan–Meier analysis was used to generate a survival curve to compare subgroup survival. Using the immunoscore website (https://bioinformatics.mdanderson.org/estimate/disease.html), we immunoscore all genes, filter for differential genes, and locate intersections with differential genes based on risk score. Analysis of the Kyoto Encyclopedia of Genes and Genomes (KEGG), Disease Ontology (DO), and Gene Ontology (GO) for functional annotation of DEGs based on the immunoscore and risk score. These DEGs were also used for analysis of the correlation between genes and immune cells and immune function.

## Results

### Identification of fatty acid metabolism related MicroRNAs

**FIGURE 1:**
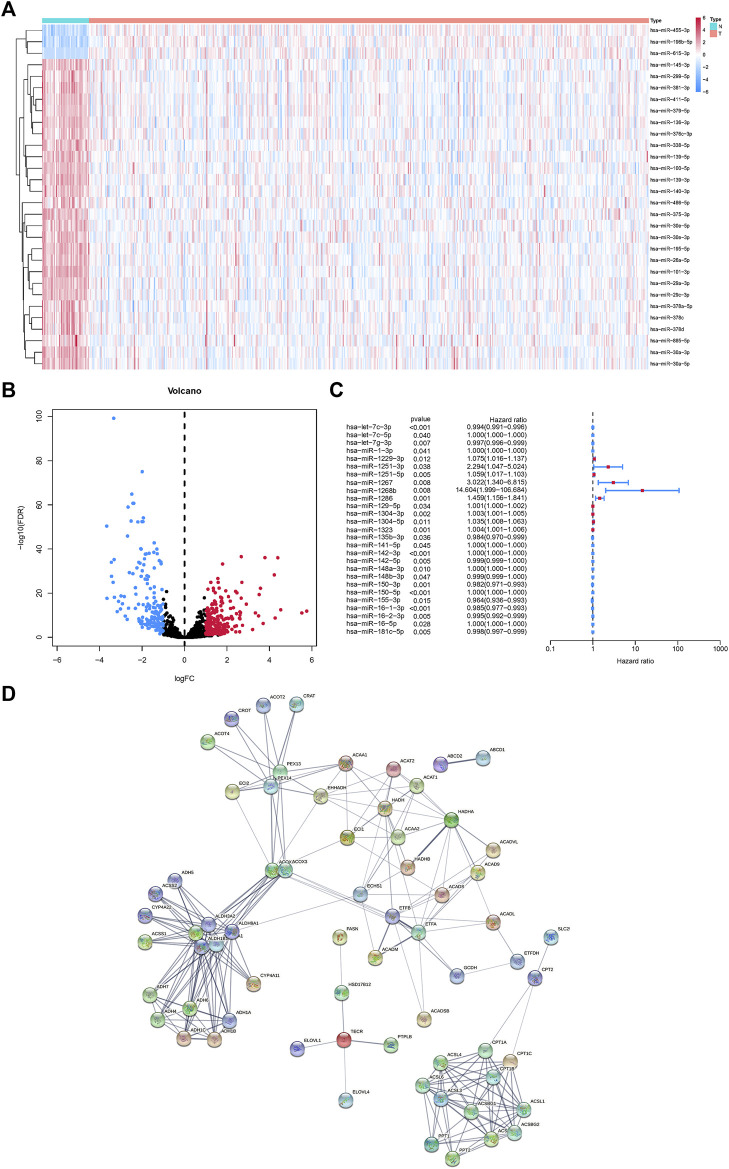
MicroRNAs associated with fatty acid metabolism in the HNSCC and matched normal tissues. **(A)**Heatmap of fatty acid metabolism-related microRNAs. **(B)** Volcano map of fatty acid metabolism-related microRNAs. **(C)** Univariate Cox regression analysis of fatty acid metabolism-related microRNAs. **(D)** Fatty acid metabolism-related microRNAs interactions revealed by PPI.

MicroRNAs related to fatty acid metabolism were extracted according to TargetScan, and the top 30 genes were displayed by heatmap (Figure 1A). Demonstration of up-and down-regulated microRNAs by volcano plot (Figure 1B). Using univariate Cox analysis, 27 microRNAs related to fatty acid metabolism were screened and shown as forest maps (Figure 1C). In addition, using the STRING database (https://string-db.org/), a protein-protein interaction (PPI) network of DEGs was constructed to reveal the interactions of the 92 metabolism-related genes (Figure 1D).

### Fatty acid metabolism related MicroRNAs based risk score construction

**FIGURE 2:**
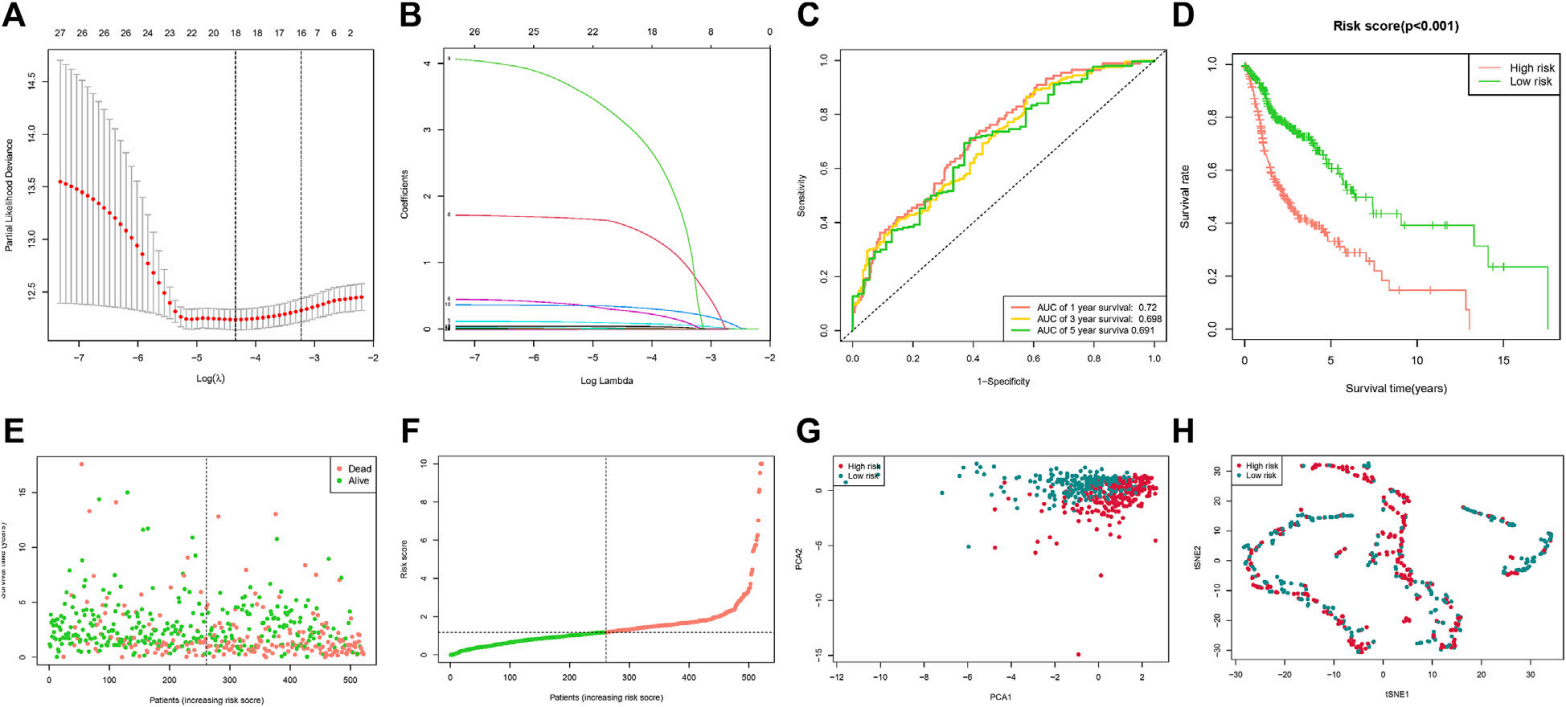
Risk score based on fatty acid metabolism-related microRNAs. **(A)** LASSO analysis with 10-fold cross-validation identified fatty acid metabolism-related microRNAs. **(B)** Coefficient profile plots of fatty acid metabolism-related microRNAs. **(C)** The ROC curves of the low-risk and high-risk groups of the risk score. **(D)** Survival curves of the low-risk and high-risk groups of the risk score. **(E)** Risk score of the HNSCC patients. **(F)** Survival comparison of the low-risk and high-risk groups in HNSCC patients. **(G)** PCA plot of the low-risk and high-risk groups. **(H)** t-SNE plot of the low-risk and the high-risk groups.

**FIGURE 3:**
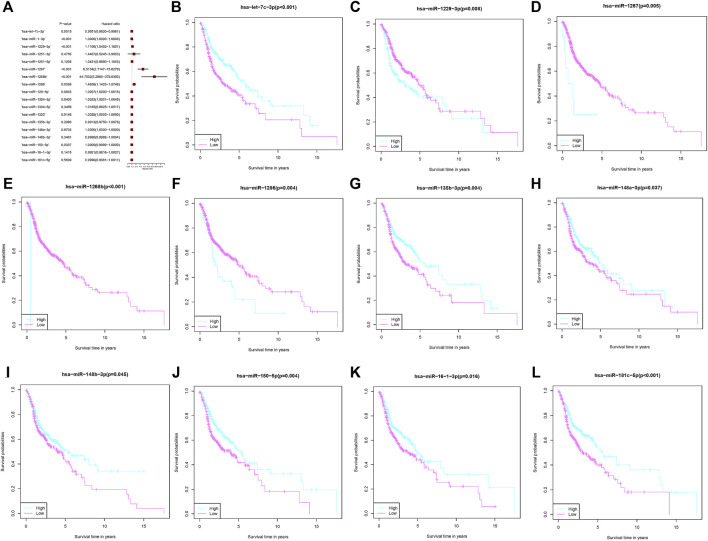
MicroRNAs associated with fatty acid metabolism that comprise the risk score. **(A)** Multivariate Cox regression analysis of fatty acid metabolism-related microRNAs. **(B–L)** The overall survival comparison between the high- and low-risk score subgroups with regard to 11 fatty acid metabolism-related microRNAs.

These 27 microRNAs were screened using lasso regression, and a total of 18 microRNAs (hsa-let-7c-3p, hsa-miR-1-3p, hsa-miR-1229-3p, hsa-miR-1251-3p, hsa-miR-1251-5p, hsa-miR-1267, hsa-miR-1268b, hsa-miR-1286, hsa-miR-129-5p, hsa-miR-1304-3p, hsa-miR-1304-5p, hsa-miR-1323, hsa-miR-135b-3p, hsa-miR-148a-3p, hsa-miR-148b-3p, hsa-miR-150-5p, hsa-miR-16-1-3p, hsa-miR-181c-5p) were filtered (Figures 2A,B). The risk score was constructed by these 18 microRNAs. The results of the ROC curve and survival curve showed the risk score can better differentiate the prognosis of HNSCC patients (Figures 2C,D). The distribution of risk scores and survival times were compared between the low-risk and high-risk groups. (Figures 2E–H). In addition, survival analysis of these 18 genes demonstrated that 11 of them (hsa-let-7c-3p, hsa-miR-1229-3p, hsa-miR-1267, hsa-miR-1268b, hsa-miR-1286, hsa-miR-135b-3p, hsa-miR-148a-3p, hsa-miR-148b-3p, hsa-miR-150-5p, hsa-miR-16-1-3p, hsa-miR-181c-5p) distinguished between HNSCC patient risk categories well (Figure 3).

### Construction of a nomogram based on risk score and clinical characteristics

**FIGURE 4:**
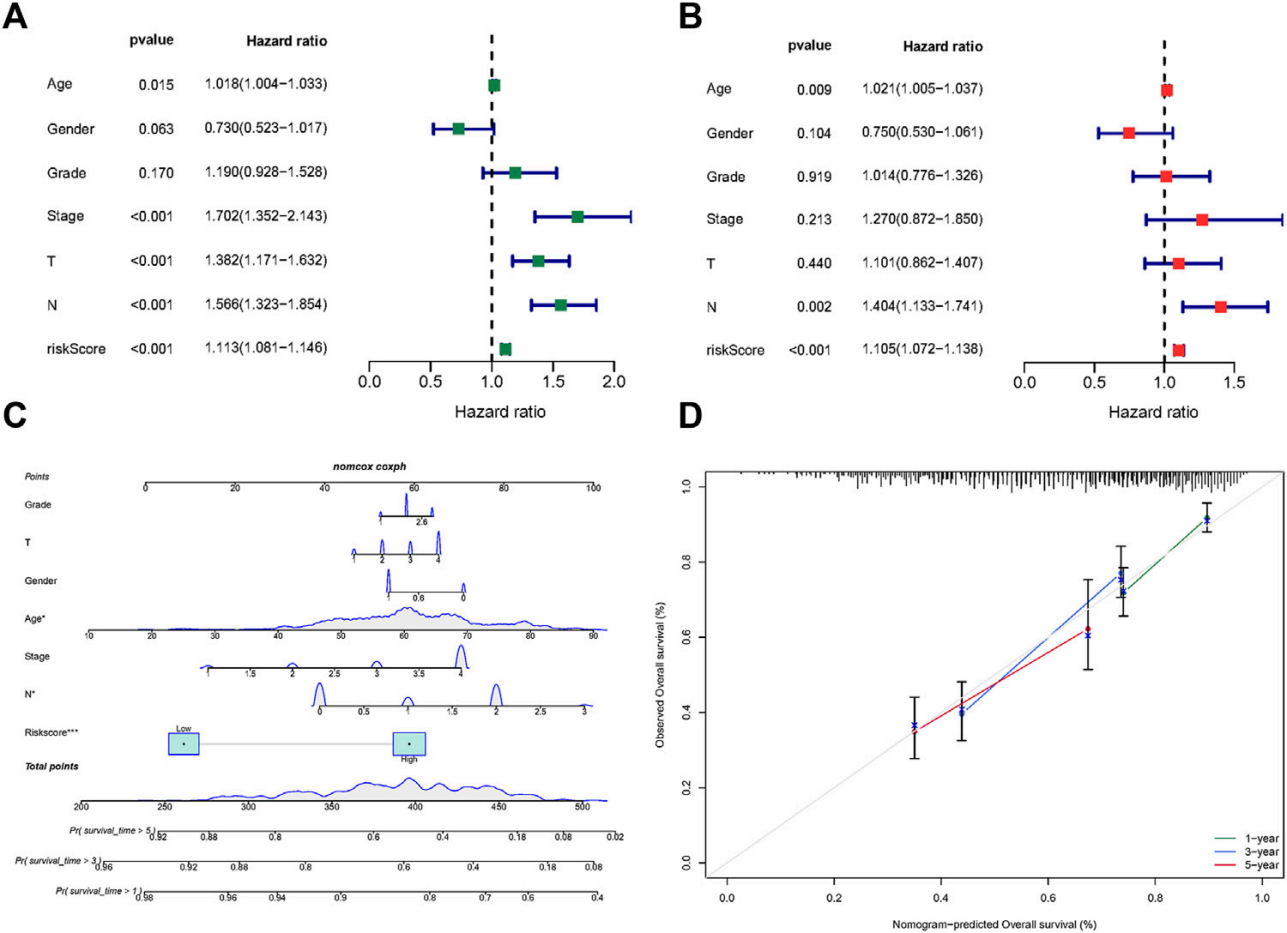
Risk score based fatty acid metabolism-related microRNAs. **(A)** Univariate Cox analysis of risk factors. **(B)** Multivariate Cox analysis of risk factors. **(C)** The nomogram integrated the risk score, age, gender, grade, and stage to predict OS. **(D)** The calibration curves for nomogram.

The results of univariate Cox regression analysis indicated age, stage (T, N) and risk score are risk factors for HNSCC patients (Figure 4A). Moreover, multivariate Cox regression analysis revealed that risk score, age, and tumor stage (N) are independent prognostic risk factors for HNSCC patients (Figure 4B). Based on age, gender, grade, stage, and risk score, a nomogram was developed (Figure 4C), and the calibration curve indicates that the Norman plot provides a better fit (Figure 4D).

### Enrichment and immune function correlation analysis based on intersection genes

**FIGURE 5:**
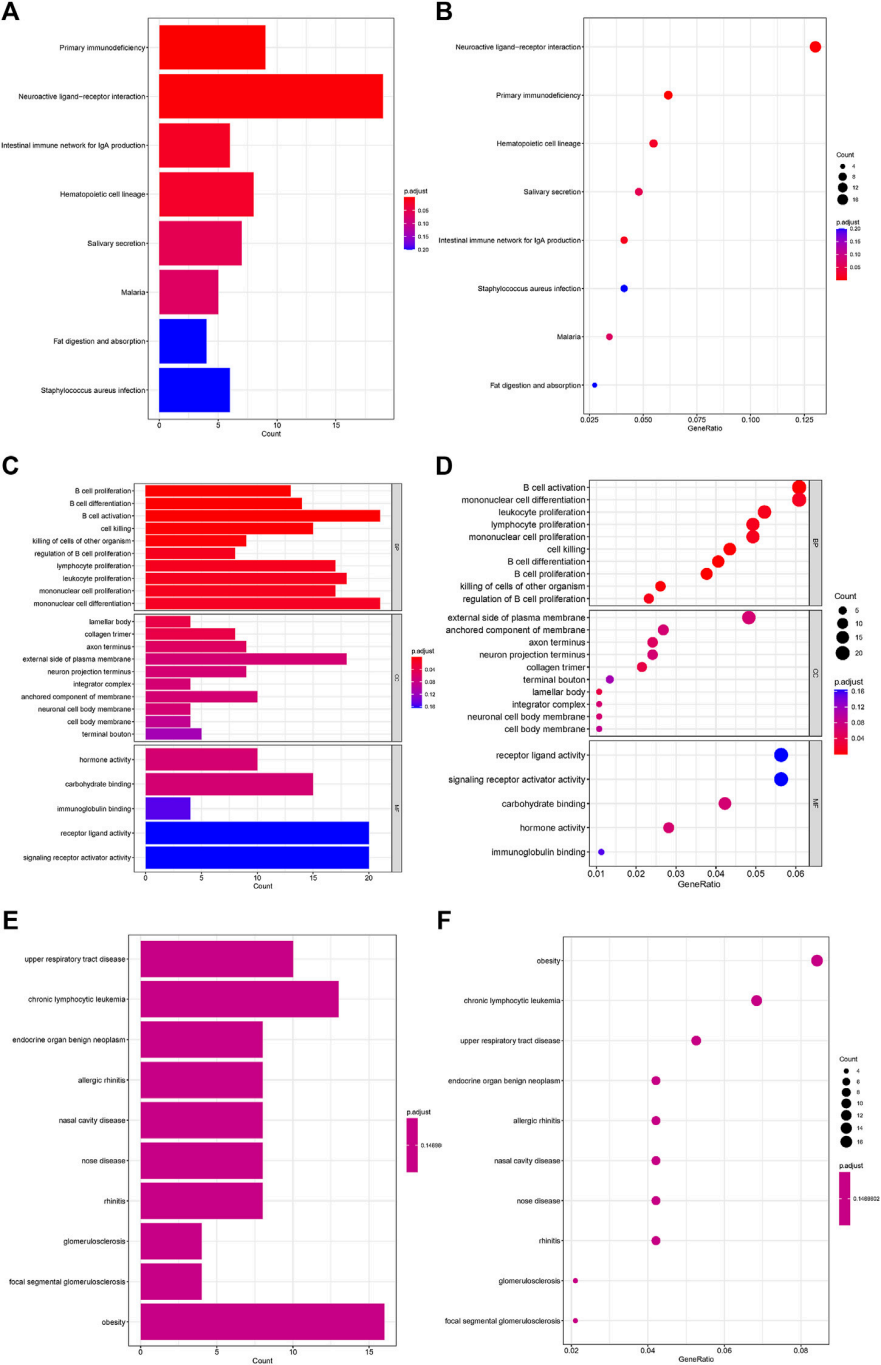
Functional enrichment analysis of intersection genes. **(A,B)** KEGG enrichment analysis. **(C,D)** GO enrichment analysis. **(E,F)** DO enrichment analysis.

**FIGURE 6:**
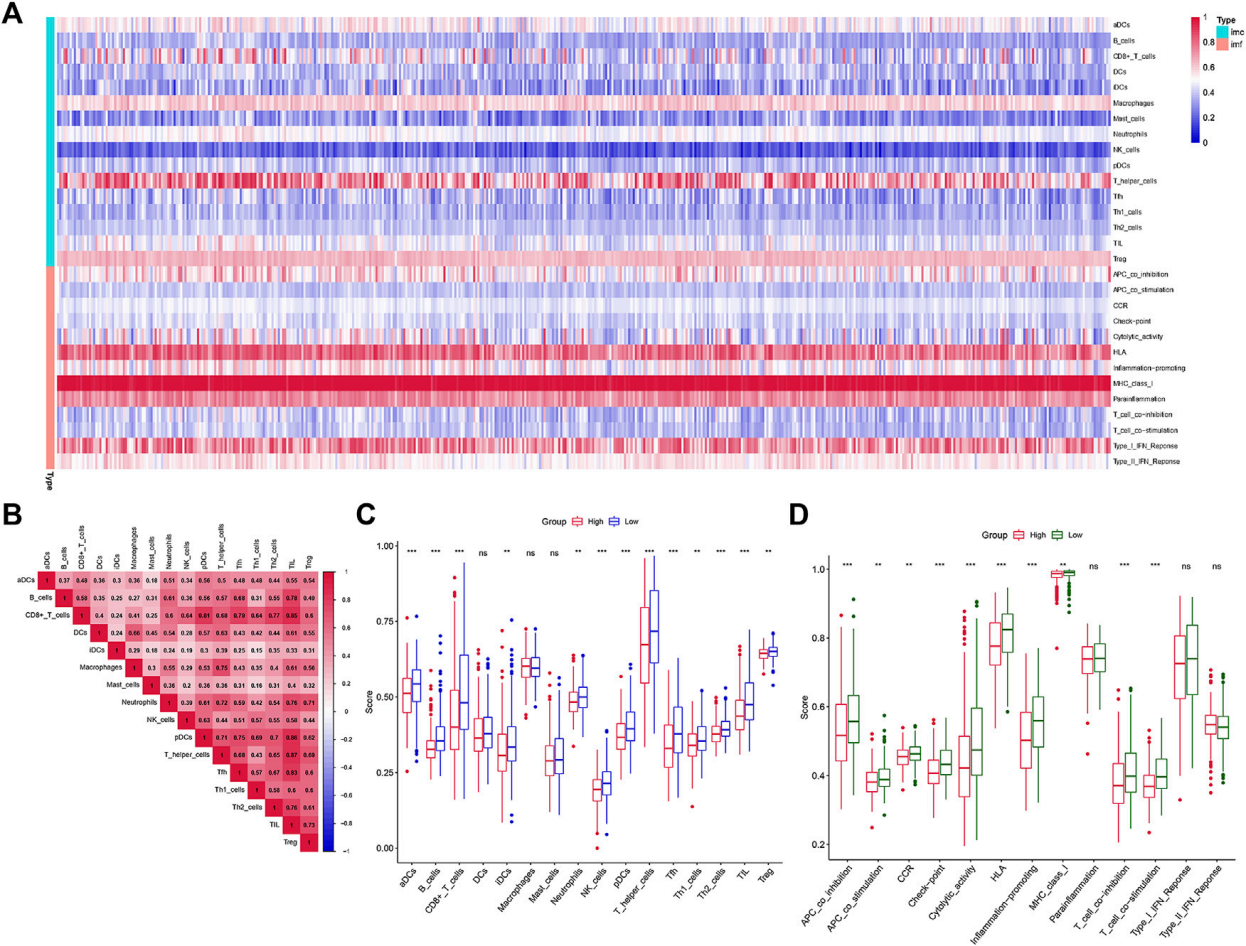
Immune function analysis. **(A,B)** The heatmap of immunocorrelation analysis. **(C,D)** Immune differential analysis and functional differential analysis between the high- and low-risk score subgroups.

**FIGURE 7:**
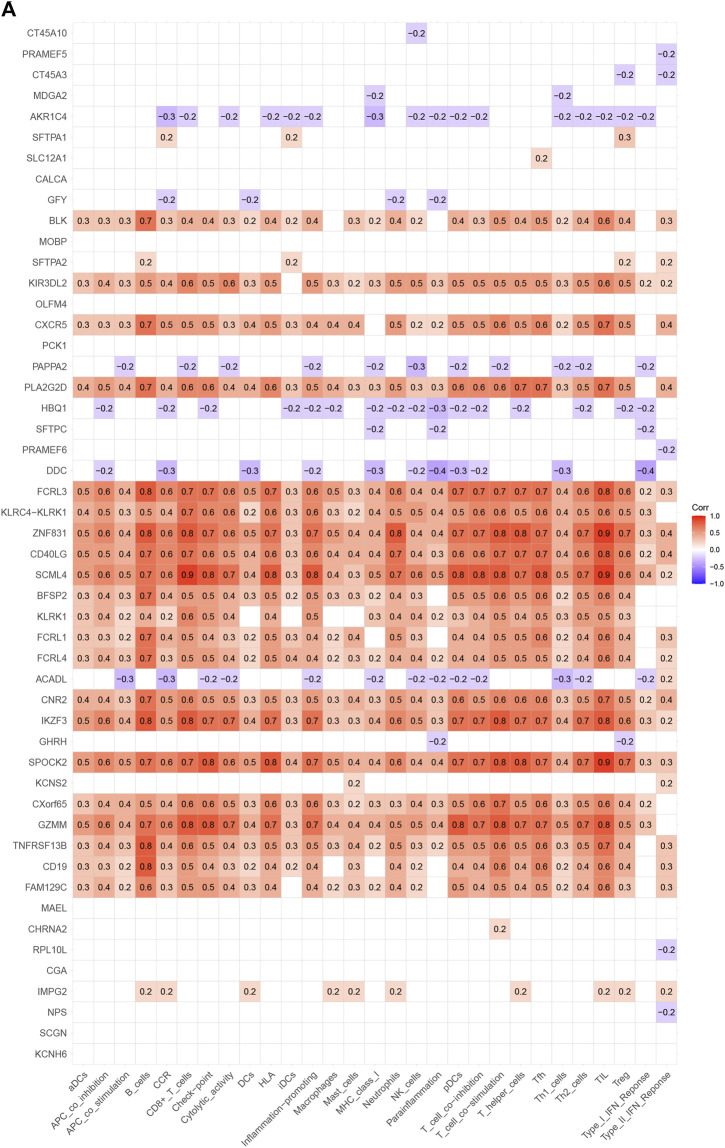
Correlation analysis of genes with immune cells and immune function in HNSCC.

Analysis of the Kyoto Encyclopedia of Genes and Genomes (KEGG), the Disease Ontology (DO), and the Gene Ontology (GO) for functional annotation of DEGs using intersection genes between immunoscore and risk score. According to the KEGG analysis, the intersection genes were mostly enriched in “Primary immunodeficiency,” “Neuroactive ligand-receptor interaction,” “Intestinal immune network for IgA production,” “Hematopoietic cell lineage,” “Salivary secretion,” “Malaria,” “Fat digestion and absorption,” and “Staphylococcus aureus infection” (Figures 5A,B). According to the GO analysis, the intersection genes were mostly enriched in “B cell proliferation”, “B cell differentiation”, “B cell activation”, “cell killing”, “killing of cells of other organism”, “regulation of B cell proliferation”, “lymphocyte proliferation”, “leukocyte proliferation”, “mononuclear cell proliferation”, “mononuclear cell differentiation”, “lamellar body”, “collagen trimer”, “axon terminus”, “external side of plasma membrane”, “neuron projection terminus”, “integrator complex”, “anchored component of membrane”, “neuronal cell body membrane”, “cell body membrane”, “terminal bouton”, “hormone activity”, “carbohydrate binding”, “immunoglobulin binding”, “receptor ligand activity”, “signaling receptor activator activity” (Figures 5C,D). Finally, according to the DO analysis, the intersection genes were mostly enriched in “upper respiratory tract disease”, “chronic lymphocytic leukemia”, “endocrine organ benign neoplasm”, “allergic rhinitis”, “nasal cavity disease”, “nose disease”, “rhinitis”, “glomerulosclerosis”, “focal segmental glomerulosclerosis”, “obesity”, “seminoma”, “overnutrition” (Figures 5E,F). To demonstrate the relationship between intersection genes and immunological function, the immunocorrelation analysis (Figures 6A,B), immunodifferential analysis (Figures 6C,D), and gene and immune cell correlation analysis (Figure 7) were constructed using intersection genes.

## Discussion

The reprogrammed metabolism of tumor cells results in changed metabolite levels in the tumor ecotone. Current research focuses on the effect of these metabolic modifications on the activity of immune cell compartments in the microenvironment. Consequently, it is essential to study and identify the multiple potential metabolic targets that impact tumor development. Thus, metabolic imbalance and transformation are regarded as hallmarks of cancer. Cancer cells have a very dynamic metabolism and continually alter how they use nutrients as fuel in and around the tumor (Elia et al., 2021). Reprogramming metabolic pathways enables cancer cells to survive and multiply in an environment deficient in nutrients (Wu et al., 2019). Aerobic glycolysis, enhanced glutamine absorption, and accelerated ab initio synthesis of fatty acids are a few of these modifications. It has been demonstrated that cancer cell-derived metabolites contribute to the hostile tumor microenvironment (Anderson et al., 2017).

Current research focuses on the effect of these metabolic modifications on the activity of immune cell compartments in the microenvironment. Immune cell metabolic reprogramming is a novel cancer characteristic that alters immune cell function by interfering with critical transcriptional and post-transcriptional activation mechanisms (Pavlova and Thompson, 2016). Numerous studies have examined the substantial consequences of altered tumor cell metabolism and immunosuppression on tumor development. Immunotherapeutic research is now concentrating on how tumor cells alter the function of immune cells and seize control of immune cells in order to shield developing tumors from immunological invasion. Decades of research have been devoted to the study of cancer biology, and several characteristics that characterize the growth of cancer have been identified. The substantial influence of cancer cell metabolic changes and suppressive immunity on tumor development has been the subject of much investigation. Focus has been placed on the development of innovative immunotherapeutic techniques due to the immune cells’ functional manipulation and takeover by cancer cells to shield the expanding tumor from immunological invasion (Siddiqui and Glauben, 2022). As part of the tumor microenvironment, several cell types and soluble chemicals (metabolites or cytokines) play a role in supporting tumor spread (TME). Infiltrating immune cells are critical in the fight against cancer, and metabolic flipping of immune cells has been shown to alter activation, differentiation, and polarization from a tumor suppressor to an immunosuppressive phenotype. Recent immunometabolism research has focused on how polarization impacts the metabolism of tumor-associated macrophages (TAMs) (Siddiqui and Glauben, 2022). For fast energy expenditure, activated pro-inflammatory macrophages rely on glycolysis, whereas alternatively activated macrophages favor fatty acid oxidation (Biswas and Mantovani, 2012). In colon cancer, fatty acids, particularly unsaturated fatty acids, drive myeloid cells of bone marrow origin to develop into a m2-like phenotype with a potent inhibitory potential, according to previous research (Wu et al., 2019).

Fatty acid synthesis is an important cellular process that makes lipids, which can then be used to make membranes or ATP. However, lipid metabolism is a sign that a disease is getting worse, and it may be very important for some types of cancer to get worse. Increased quantities of fatty acids generated and accumulated by several solid tumors have been documented to provide a tumor microenvironment rich in fatty acids. In malignancies of the prostate, colon, ovary, liver, and lung, genes involved in adipogenesis have been identified as being activated (Swinnen et al., 2006). There is additional evidence that the propensity for fatty acid metabolism alters in breast cancer subtypes with differential RARRES1 expression. In breast cancer, the state of hormone receptors coincides with alterations in lipid metabolism (Camarda et al., 2016; Yamashita et al., 2017).

Previous study has shown a potential relationship between fatty acid metabolism and tumor development and patient prognosis. However, fatty acid metabolism-related microRNAs in HNSCC and how they affect a patient’s prognosis is not clear. The results of this paper revealed that microRNAs involved in fatty acid metabolism were considerably altered in HNSCC tumor tissues compared to healthy controls. Through univariate Cox regression analysis, we found that 27 of these differentially expressed microRNAs are linked with HNSCC patient prognosis (*p* < 0.05). We utilized lasso regression to identify microRNAs that have great strongly linked with the prognosis of HNSCC patients and used them to create risk scores. The results of ROC and survival curve data demonstrated that the risk score has a better prognosis distinction for HNSCC patients. Furthermore, survival analysis of these microRNAs revealed that 11 of them (hsa-let-7c-3p, hsa-miR-1229-3p, hsa-miR-1267, hsa-miR-1268b, hsa-miR-1286, hsa-miR-135b-3p, hsa-miR-148a-3p, hsa-miR-148b-3p, hsa-miR-150-5p, hsa-miR-16-1-3p, hsa-miR-181c-5p) discriminated effectively between HNSCC patient risk groups. However, the function of these genes in HNSCC remains unclear. A recent study found that PSMC2 promotes gastric cancer progression by targeting hsa-let-7c-3p to increase the level of RPS15A, which then activates the mTOR pathway (Liu et al., 2022). This indicates that hsa-let-7c-3p may regulate biological processes such as cancer and development. Previous study found human mirtronic microRNAs are reported to be differently regulated in several cancer cell lines and tumors. In particular, hsa-miR-1229-3p was specifically increased in metastatic pancreatic and gastric cancer cell lines (Butkyte et al., 2016). Although studies of hsa-miR-1268b in tumors have not yet been reported, hsa-miR-1268b can be involved in a-linolenic acid metabolism (Gao et al., 2018). Since a-linolenic acid metabolism can regulate sugar, lipid, and protein metabolism, we theorized that hsa-miR-1268b may affect the tumor microenvironment by regulating fatty acid metabolism. Despite the fact that hsa-miR-135b-3p can identify human ovarian cancer tissue from normal tissue (Wang et al., 2014), its role in controlling the metabolism of the tumor microenvironment is unknown. Prior research demonstrated that hypoxia downregulation of hsa-miR-148a-3p led to the overexpression of its two target genes, ITGA5 and PRNP, which was a factor in colorectal cancer patients’ tumor development and poor prognosis (Nersisyan et al., 2021). Our research demonstrates that hsa-miR-148a-3p properly distinguishes the prognosis of HNSCC patients. In addition, a screening of differentially expressed miRNAs in gastric cancer showed hsa-miR-148a-3p, hsa-miR-148b-3p, and hsa-miR-363-3p as having the greatest number of target genes, three microRNAs were significantly overrepresented in numerous cancer-related pathways, including the “Wnt signaling route,” the “MAPK signaling pathway,” and the “Jak-STAT signaling pathway” (Luo et al., 2015). Overexpression of hsa-miR-150-5p accelerated the passage of human cervical cancer cells from G0/G1 to S phase, resulting in a considerable increase in cell proliferation (Oboshi et al., 2020). In addition, the overexpression of hsa-miR-150-5p was linked to an increased risk of Chronic lymphocytic leukemia (Casabonne et al., 2020). Previous study suggested that hsa-miR-181c-5p may participate in DNA methylation control in HPV-16-induced HNSCC(Sannigrahi et al., 2018). Our findings indicate that hsa-miR-181c-5p is a regulatory gene associated with fatty acid metabolism and predicts the prognosis of HNSCC. We speculated hsa-miR-181c-5p may alter the prognosis of HNSCC by influencing DNA methylation associated with fatty acid metabolism. Until now, the neoplastic and metabolic roles of hsa-miR-1267, hsa-miR-1286, and hsa-miR-16-1–3p are unknown.

In conclusion, acid metabolism-related microRNAs may play a role in the progression of HNSCC. Although there has been much research on HNSCC related microRNAs, there has been little on its reprogramming of fatty acid metabolism and microRNAs that regulate fatty acid metabolism. Moreover, the enrichment analysis of KEGG, GO, and DO revealed that interconnection genes were predominantly in fatty acid metabolism-related pathways, including fat digestion and absorption, obesity, and overnutrition, as well as immune-related pathways, including B cell proliferation, B cell differentiation, B cell activation, cell killing, killing of other organisms’ cells, regulation of B cell proliferation, lymphocyte proliferation, leukocyte proliferation, mononuclear cell proliferation, and regulation of B cell proliferation. So, in the future, more emphasis should be directed to HNSCC metabolic reprogramming, particularly changes in fatty acid metabolism and the accompanying microRNA regulatory network, which may reveal new immunotherapeutic targets for the treatment of HNSCC.

## Data Availability

The original contributions presented in the study are included in the article/Supplementary Material, further inquiries can be directed to the corresponding authors.
